# The food industry’s role in influencing consumer demand for healthy and unhealthy food: perspectives from Australian food companies

**DOI:** 10.1017/S1368980026101943

**Published:** 2026-01-30

**Authors:** Josephine Marshall, Jasmine Chan, Sally Schultz, Sarah Dean, Cliona Ni Mhurchu, Gary Sacks

**Affiliations:** 1 Global Centre for Preventive Health and Nutrition, Institute for Health Transformation, Faculty of Health, Deakin University, Geelong, Australia; 2 Department of Epidemiology & Biostatistics, School of Population Health, University of Auckland, Auckland, New Zealand; 3 The George Institute for Global Health, Sydney, Australia

**Keywords:** Food company, Nutrition, Consumer demand, Food environment, Stakeholder perspectives

## Abstract

**Objective:**

This study sought to explore how food company representatives perceive the food industry’s role in responding to and driving consumer demand for healthy and unhealthy foods.

**Design:**

Semi-structured interviews were conducted in 2022 by 2–3 researchers to explore food company representatives’ perspectives related to consumer demand for healthy and unhealthy food. Detailed field notes, including verbatim quotes, were recorded, and the data were analysed thematically.

**Setting:**

This study was part of a government-funded 12-month intervention programme to assess the impact of tailored support for food companies on company nutrition-related policies and practices.

**Participants:**

Thirty-two food company representatives from thirteen large food and beverage manufacturers in Australia.

**Results:**

Six themes were identified. Company representatives acknowledged that manufacturers actively shaped demand for both healthy and unhealthy foods. Healthy reformulation and aspects of nutrition labelling were constrained by anticipated consumer resistance, while demand for ‘less healthy’ products was driven by non-health attributes such as taste, comfort and affordability. Internal company marketing teams held significant influence regarding product development, promotion and labelling. Supermarkets were perceived as shaping demand via their marketing strategies. The competitive landscape, driven by the pursuit of market share, was seen to fuel an ongoing cycle of promotion of ‘less healthy’ products.

**Conclusions:**

Food companies acknowledge playing an active role in influencing consumer demand for healthy and unhealthy food and beverages. A whole-of-system response, including changes in government regulation and practice change by the food industry, is needed to drive stronger action and accountability from food companies to support healthier diets.

Australian retail food environments are dominated by unhealthy packaged foods that are heavily marketed^(1–5)^. The unhealthy state of Australian food retail environments is reflected in Australians’ dietary intake, with low consumption of healthy food groups, and discretionary food (typically high in added sugar, salt and/or saturated fat) making up over a third of adults’ energy intake^(6,7)^. Improving the healthiness of food environments has been identified by the Australian government as a key area of focus as part of efforts to improve population diets and prevent obesity. Current strategies and initiatives introduced by the government include the development of the National Preventive Health Strategy (2021–2030), the National Obesity Strategy (2022–2032), Healthy Food Partnership reformulation targets and Health Star Rating (HSR) front-of-pack labelling scheme^(8–11)^. Recommended policy actions for the Australian government include introducing legislation to reduce the exposure of children to unhealthy food marketing and improvements to nutrition labelling, including mandating the adoption of the HSR scheme^(12)^. Currently, there is an absence of government regulatory action to support these priorities, meaning nutrition-related policy action relies heavily on food and beverage companies (herein referred to as food companies’) taking voluntary action to improve their nutrition-related policies and practices.

Whilst some food companies in Australia have taken steps to improve some of their nutrition-related practices, the sector is falling far short of best practice recommendations^(13–15)^. In fact, there is strong evidence that food companies have actively resisted efforts to improve the healthiness of food environments and address their harmful impact on diets and population health^(16–20)^. A common argument used by food companies is that their product development and marketing tactics merely respond to consumer demand^(21)^. However, this claim contradicts research showing that many Australians are trying to eat healthily and would support food companies taking a broad range of actions to improve nutrition and the healthiness of food environments^(22–26)^. Moreover, studies have shown that, over time, food company processing, packaging and marketing techniques have shaped consumer preferences and increased demand for unhealthy food^(27,28)^. Despite this, common industry narratives based on principles of consumer sovereignty continue to appear in industry submissions on government policies and initiatives, as well as in industry-driven media reports on food policy, and may have contributed to limited policy action in this area^(29–31)^.

Understanding how food companies perceive their role in shaping consumer demand for healthy and unhealthy food is critical for informing evidence-based policies that hold companies accountable for their influence on population diets. To date, beyond analyses of publicly available information, no studies have directly examined food companies’ understanding of how they influence consumer demand for food with respect to healthiness. This study aimed to explore how food company representatives perceive the food industry’s role in responding to and driving consumer demand for healthy and less healthy foods.

## Methods

### The REFORM study

The data reported in this paper were collected as part of the REFORM study, a government funded 12-month intervention programme to assess the impact of tailored support to food and beverage manufacturers and quick service restaurants on company nutrition-related policies and practices^(32)^. REFORM focused on the largest packaged food and beverage manufacturers in Australia and New Zealand (> $10 million in annual retail sales revenue), identified using Euromonitor 2019 data and randomised to intervention and control groups^(33)^. The REFORM intervention involved six meetings per company, where company representatives (nominated by the company) met with the research team. The interactive meetings provided a platform where the REFORM research team shared public health perspectives, provided resources and recommendations related to nutrition policies and practices and engaged in dialogue to understand industry views. The programme covered a range of topics including healthier product reformulation and development, nutrition labelling, consumer nutrition trends for healthy food, company nutrition policies and nutrition-related corporate sustainability reporting. This study is based on data from Australian companies.

### Characteristics of participating companies and representatives

Forty-seven companies were invited to be part of the REFORM programme via email and/or phone calls, with thirteen companies with head offices in Australia accepting and participating in the programme. Five companies were classified as large (≥ $250 million annual retail sales revenue), and eight companies were classified as medium ($10 m – $250 million). Participating companies produced and sold a diverse range of healthy and unhealthy packaged food and beverages, including sweet and savoury snacks, dairy products, frozen or canned fruit and vegetables, frozen savoury foods (including frozen pies, frozen crumbed or battered fish), muesli/cereal/snacks and meal bases/sauces. Based on previously published information^(1)^, the healthiness of product portfolios was assessed using the HSR system^(11)^, which rates a product’s overall nutritional profile from 0·5 to 5 stars (with 0·5 increments), where 0·5 stars is the least healthy and 5 stars is the most healthy. Five companies had an average HSR of 3·5 stars or higher, four companies ranged between 2·5 and 3·5 stars and four companies had an average below 2·5 stars^(1)^. Table 1 summarises the number of food company representatives that participated in the study, the types of roles they held in the company and the data related to the healthiness of the company’s product portfolio. A total of thirty-two company representatives participated, with a minimum of 1 and a maximum of 7 from each company.

**Table 1.** Description of companies and company representatives that participated in the meeting focused on ‘consumer nutrition trends and demand for healthy food’, March–June 2022

Company ID	Average HSR of product portfolio^*^	% of products with HSR above ≥ 3·5 stars^*^	No. of company representatives who participated in meetings	Type of roles of participating company representatives
10–023	< 2·5	0–25 %	2	Manager – Product Development (1), Food Technologist (1)
10–024	> 3·5	76–100 %	2	Manager – Nutrition (1), Manager – Product Development (1)
10–025	< 2·5	51–75 %	1	Manager – Regulatory Affairs (1)
10–029	2·5–3·5	51–75 %	3	Manager – Regulatory Affairs (2), Nutritionist (1)
10–032	> 3·5	76–100 %	1	Manager – Research and Development (1)
10–033	< 2·5	0–25 %	1	Food technologist (1)
10–041	2·5–3·5	51–75 %	2	Executive Manager – Marketing (1), Manager – Product Development (1)
10–043	> 3·5	76–100 %	7	Executive Manager – Research and Development (1), Marketing roles (6)
10–049	> 3·5	76–100 %	3	Manager – Marketing (1), Manager – Product Development (2)
10–050	> 3·5	76–100 %	2	Manager – Research and Development (1), Nutritionist (1)
10–051	< 2·5	0–25 %	3	Manager – Product Development (1), Manager – Research and Development (1), Manager – Regulatory Affairs (1)
10–054	2·5–3·5	26–50 %	2	Executive Manager – Product Development (1), Manager – Regulatory Affairs (1)
10–056	2·5–3·5	0–25 %	1	Manager – Research and Development (1)

HSR, Health Star Rating.

* Based on data provided by the George Institute for Global Health for this project, including data from the State of the Food Supply Report: A Five-Year Review. (1)

Although the scope of these roles varied across companies, they typically encompassed the following functions: Product Development positions were responsible for the strategic and operational aspects of product innovation and reformulation including market and financial feasibility; Research and Development roles were responsible for the technical aspects of product development, including ingredient sourcing and the technical systems and processes used in new and reformulated products; Food Technologists played a hands-on role in ensuring their technical feasibility; Nutritionists provided guidance such as establishing nutrient guardrails for product development; and Regulatory Affairs ensured compliance with policies and legislation, including those governing health and nutrient content claims. Most roles influenced nutrition-related actions to varying degrees, with three executive managers holding decision-making authority within their domains.

### Data collection

**Figure 1.**
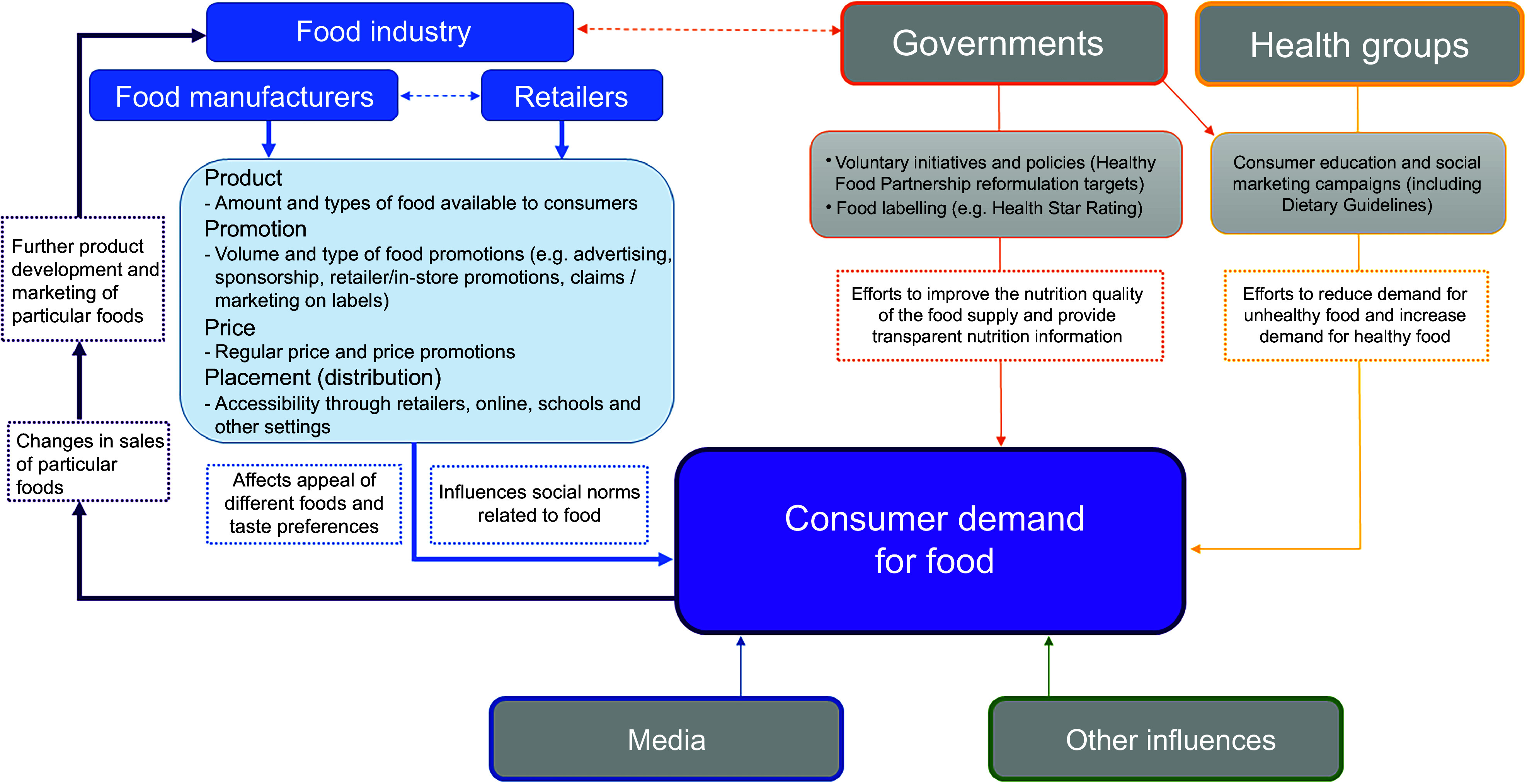
Modified version of the conceptual framework of food systems for diets and nutrition developed by the High Level Panel of Experts on Food Security and Nutrition (HLPE).

The data reported in this study were collected during the fourth meeting in the series of six, on the topic of ‘consumer nutrition trends and demand for healthy food’. The research team had limited (GS) or no (JM, JC, SS, SD and CNM) prior relationship with the company representatives before the series of meetings. Each of the thirteen separate meetings on this topic involved 2–3 research team members (JM, JC and SD) and representatives from within the food company. Meetings were held online using Zoom or Microsoft Teams between March and June 2022, with an average duration of 60 min. Participants were informed of the purpose of the REFORM study and research team members introduced themselves during meetings, including their occupation and role in the study. The first part of the meeting (approx. 20 min) involved the research team providing company representatives with public health evidence related to consumer demand for healthy food and shared a modified version of the conceptual framework of food systems for diets and nutrition, developed by the High Level Panel of Experts on Food Security and Nutrition (HLPE) (refer to Figure 1)^(34)^. The HLPE framework was adapted to focus on the factors that influenced consumer behaviour and demand that related to food companies (see Appendix A for slide deck presented in the meeting). In the second part of the meeting, company representatives were asked for their views on the framework and how they perceived their company influenced consumer demand for healthy and unhealthy food (approximately 40 min). See Appendix B for the semi-structured interview guide.

Detailed written field notes, including verbatim responses, were taken by a dedicated member of the research team (SD), with permission from the company representatives. Meetings were not audio-recorded, as it was considered that recording may have influenced the extent to which company representatives spoke freely^(35)^ and was not part of the intervention protocol^(34)^. Field notes were reviewed immediately after each meeting by the other 1–2 members of the research team that were present in the meeting to ensure accuracy and completeness. To maintain analytic independence and avoid potential industry influence on how findings were interpreted, company representatives were not given field notes or draft themes to review.

The study was approved by the Deakin University Human Research Ethics Committee (approval HEAG-H 236_2020). Participants consented to be involved in the REFORM study and that notes from the meeting would be used for academic publications. They were informed that companies and company representatives would not be identified in results or communications about the outcomes.

### Data analysis

Data (field notes) were managed using QSR N-Vivo (Release 1.7). Data were analysed thematically using Braun and Clarke’s six-step approach^(36)^. In step 1, field notes were read and reread by the lead author (JM), noting initial reflections. Step 2 involved field notes for two companies being coded independently by three researchers (JM, JC and SS) informed by the research questions and the conceptual framework of food systems (described above). The three researchers met to discuss initial codes and generated a coding framework. Two researchers (JM and JC) then used this coding framework to deductively code the remaining data independently, whilst remaining open to the identification of new codes. In step 3, draft themes were constructed from the codes by the first author (JM) and then presented to members of the research team (JC, SS, SD and GS) where the themes were reviewed and workshopped (step 4). Themes were refined further by the research team to ensure they accurately reflected the data and answered the research questions (step 5) and presented as results, supported by illustrative quotes (step 6). Throughout the data collection and analysis, the research team engaged in reflective practice. The research team has extensive expertise in policy research to improve population diets and public health and shares the view that a whole-of-system approach is required to improve the healthiness of the food environment, involving both private sector and government action. Immediately following each meeting, the research team met to debrief (and take notes) on the views expressed by the company representative, how these views compared and contrasted to their own public health perspectives and discussed how this may affect the interpretation of the data. Any contextual observations, including group dynamics, that may also impact data analysis were also discussed and noted. This study is reported in accordance with the Consolidated Criteria for Reporting Qualitative Research (COREQ) guidelines (see Appendix C).

### Positionality and reflexivity

The research team included five women and one man, all full-time public health researchers at universities, two professors with PhD, three associate research fellows (BNutrDiet(Hons), BNut(Hons) and BHealthSci) and one PhD candidate (BHealthSci(Hons)).

The research team adopted a critical perspective in analysing the practices of food industry actors, embedded within scholarship on the commercial determinants of health. In particular, the research team were conscious of the large body of literature documenting the wide range of strategies used by food industry actors to advance industry interests and shape public health discourse in ways that may not align with public health goals. We applied a public health lens to all aspects of programme design, implementation and data interpretation, including regular deliberative reflection amongst team members about the framing and potential motivations of food industry actors.

This study was based on data from the fourth meeting in a six-part series of interactive meetings with food companies, by which point the research team had developed in-depth knowledge of participating companies’ policies and practices. This sustained engagement allowed for the development of rapport and trust with company representatives, facilitating more open, reflective and substantive dialogue. The team remained attentive to power dynamics and the potential for strategic positioning by company representatives and critically examined claims in light of broader evidence and context.

## Results

Thematic analysis of field notes exploring company representatives’ views about the role of food companies in driving consumer demand for healthy and unhealthy products resulted in six key themes, described below.

### Theme 1: Food manufacturers acknowledge their role in creating demand for healthy and unhealthy food

Participants stated that their company’s and the broader food industry’s drive to generate revenue and profit meant that food companies not only responded to consumer demand but also actively shaped demand for both healthy and unhealthy food. Some participants noted that their companies invested heavily in understanding current consumer behaviour and in anticipating future trends. These insights were used to guide product development and marketing strategies aimed at driving demand, for example, by creating new products aligned with the latest food and nutrition trends such as high-protein food, reformulating existing products in response to perceived demand for healthier options within a category and promoting these through health or nutrition claims and other marketing tactics.

*‘A lot of the time when we are working ahead of the game and researching what products to develop, consumers can only see what they know. So, it’s on us to predict what consumers want, what future trends will be, and what they [consumers] are going to need’*. (Manager (Marketing), Company 10–043)

Participants acknowledged the tension between commercial success and public health, noting that strong sales of unhealthy food and beverages incentivised their continued development and promotion, despite potential harms to consumer health.

*‘The problem is that stuff [unhealthy food] sells, so [when unhealthy foods sell well] retailers are happy, and manufacturers are happy, and consumers think they’re happy but they’re slowly poisoning themselves’*. (Manager (Marketing), Company 10–049)

Conversely, some participants noted that growing consumer interest in nutrition created a willingness within their companies tow adopt healthier policies and practices, designed to boost demand and sales for healthier options. Some participants also described that their companies have implemented practices such as internal nutrition guidelines and targets, so even if consumer insights indicate there is a demand for less healthy products, food companies can still make decisions that support population health.

*‘We develop guidelines for sugar, salt, fat… Consumers might ask for more salt but it doesn’t mean we are going to double the amount just so we can sell more products’*. (Manager (Nutrition), Company 10–024)

Other participants, particularly from companies with less nutrition expertise, emphasised the need for clear, consistent government and public health guidelines to help food companies interpret consumer trends and insights through an evidence-based understanding of healthy food and nutrition.

### Theme 2: Anticipated consumer backlash poses a challenge to healthy product reformulation and labelling

Participants discussed how their food company was unlikely to voluntarily implement nutrition-related strategies if they expected such actions to reduce consumer demand for the product or category. Two participants discussed how previous attempts to reformulate popular products that had resulted in negative consumer feedback and declining sales, discouraged further efforts by their company to improve the healthiness of their product portfolio.

*‘Our consumers are very loyal and we’ve been stung by making changes in the past, a lot of backlash from customers’*. (Manager (Regulatory Affairs), Company 10–025)

*‘If you tell the consumer you’ve taken 30 % of the sugar out, the backlash is incredible. You’ve changed it, how dare you, people write in letters and get very fired up’*. (Executive Manager (Marketing), Company 10–041)

Some companies reported that taking a quiet, incremental approach to reformulation of less healthy products to avoid such potential consumer backlash.

*‘Sometimes you don’t even need to tell the consumer it’s healthier. Can just have our internal policies – e.g. set a ceiling on sodium and then figure out other ways to deliver the flavour to consumers that they need. So we’re still contributing overall to a community benefit’*. (Manager (Product Development), Company 10–041)

### Theme 3: Creating demand for unhealthy food based on non-health-related attributes of food

Several company representatives viewed their role in the food supply as providing broad consumer choice, including products that deliver non-health-related benefits such as ‘enjoyment’, ‘joy’ and ‘comfort’. This often resulted in the expansion of unhealthy product lines, commonly referred to as ‘premium’ or ‘indulgent’ ranges. Some participants suggested these products were intended for occasional consumption and, as such, did not see them as major contributors to unhealthy diets in Australia. Furthermore, these indulgent ranges were typically excluded from reformulation initiatives or held to less stringent nutrition criteria, based on the perception that their indulgent positioning exempted them from needing to be healthier.

*‘We do have different nutrition criteria based on the product category, it’s not a one size fits all [approach to product development]…[the nutrient profile is] driven by how often a product would be consumed’*. (Manager (Research and Development), Company 10–032)

Another non-health benefit discussed by participants related to food acceptability and affordability. In this context, participants described that their food company responded to perceived demand from families for affordable food options they know children will eat, even if they are not necessarily healthy.

*‘With the cost of living going up, people are looking to buy something that they know the kids will eat, as opposed to choosing a healthy product they may not eat’*. (Manager (Research and Development), Company 10–032)

### Theme 4: Marketing teams shape consumer demand for healthy and unhealthy foods

Participants consistently noted the power imbalance within their company, whereby marketing teams have a substantial influence in driving their company’s response to nutrition-and health-related trend, compared with other teams.

*‘The marketing and innovation teams will work towards finding any gap into the market that we can then create demand from. That’s their job. It’s in their job description. It’s a function of the market and doing business in the food industry’*. (Food Technologist, Company 10–033)

Some spoke about the misalignment between the marketing teams understanding of health and nutrition and the views of the company nutrition experts. In particular, the marketing teams often focused on the latest consumer trends or ‘health halos’, which were often not aligned with public health recommendations.

*‘Around the whole discretionary products having health halos and carrying health claims…‘Marketing’ just want to slap on a million claims, and we [the nutrition team] are looking to develop policy for [responsible use of claims]’*. (Manager (Nutrition), Company 10–024)

Participants indicated that marketing teams also had a substantial influence over the adoption of the HSR initiative across their portfolio, directly affecting consumers’ ability to make an informed choice about the food they purchase, particularly in less healthy categories.

*‘We do put the HSR [Health Star Rating] on all our [healthier products] but in the [less healthy product category], it’s still not being adopted as it’s an indulgent product and ‘Marketing’ aren’t really keen on that’*. (Food Technologist, Company 10–033)

In order for companies to rebalance the power dynamic between marketing teams and those advocating for healthy product portfolios, participants spoke about the importance of involving marketing teams on discussions about nutrition on a routine basis.

*‘[Our company] does have health on our radar, and that we need to be working to improve health. While we definitely have that in ‘R&D’ [research and development] and ‘Regs [regulation] and Compliance’, I think ‘Sales and Marketing’ probably need to be more involved, because they’re the ones dealing with the customers or retailers – they need to be on board with what we want and what our vision is for health’*. (Senior Manager (Product Development), Company 10–051)

### Theme 5: Supermarkets placement and promotion tactics shape consumer demand

Participants indicated that the two largest supermarket retailers in Australia were particularly influential on how food companies shaped consumer demand. Supermarkets were described as the ‘gatekeepers’ that determined what products manufactured by food companies were made available and visible to consumers, resulting in greater demand for those products.

*‘The retailer has the final say [about what products are stocked] and often the healthier foods aren’t the most popular foods. And even if we want those options for consumers, retailers might not agree with our view and consequently those healthier choices get crossed out [discontinue being stocked] by the retailer’*. (Manager (Regulatory Affairs), Company 10–029)

Supermarket promotional tactics, especially the use of price promotions on unhealthy foods compared to healthy options, were identified by participants as a key driver of consumer demand for unhealthy products.

*‘At end of the day consumers do want to eat healthier, but products that are heavily discounted aren’t in the category of ‘better for you’.* (Executive Manager (Product Development), Company 10–054)

Conversely, retailers were also seen, in some instances, as helping to set the tone on health and nutrition and generate consumer demand for healthy foods, particularly through their communication, promotion and placement strategies.

*‘The likes of retailers like [name of large retailer] have an emphasis on health. They’ve got ads on TV, using HSR to communicate better choices, and they’re putting pressure on us, and all their industry partners to come to the party [take action on health and nutrition]’*. (Manager (Regulatory Affairs), Company 10–025)

### Theme 6: The competitive landscape

Participants indicated that consumer demand for unhealthy food and beverages was being driven by a food retail environment characterised by food companies seeking competitive advantage and market share. In the pursuit of increased sales, companies may introduce and promote less healthy products within a category. Then to avoid losing competitive ground, other companies often follow suit, collectively reshaping the health profile of the category. For example, one participant discussed an observed shift in the yogurt category from healthy, plain-style yogurts to dairy products with a nutrient profile that resembles desserts, which in turn has resulted in increased consumer demand for these less healthy options.

*‘You can see with brands like [competitor company], that they’ve moved away from yoghurt being healthy, to now being closer to dairy desserts and indulgent ice cream. And now there is a whole new category and demand space created for indulgent yoghurts that really are just glorified bags of sugar. I think it speaks to demand creation’*. (Food Technologist, Company 10–033)

Regarding ways to improve consumer demand for healthier food, participants suggested that a whole-of-industry approach was required.

*‘There needs to be collaboration between industry [food manufacturers] and retailers… but unless the whole industry is working in this space [healthier], there’s no prizes for being the healthiest’*. (Manager (Regulatory Affairs), Company 10–025)

Participants suggested retailer policies or government regulation could be introduced to facilitate an even playing field by reducing the potential competitive disadvantage for producing healthier products or displaying the HSR. Opinions on support for government regulation and mandatory policies to create a level playing field differed depending on the initiative, with most (but not all) company representatives expressing support for the government to make the HSR mandatory.

*‘[We] would like to see HSR as mandatory, because then it’s a level playing field. This plays into consumers’ behaviour then too because everything is displayed properly on shelf’*. (Nutritionist, Company 10–029)

## Discussion

This study found that representatives from thirteen major food companies in Australia acknowledged that the food industry actively contributed to driving consumer demand for both healthy and unhealthy food. Participants spoke about how a consequence of food companies’ profit-seeking imperative is that product healthiness is often a secondary consideration and may be compromised by their efforts to produce palatable and popular products. The marketing teams were seen as having considerable influence on consumer demand through strategic business decisions regarding new product development, labelling and promotional strategies. Opportunities for the food industry and other sectors, including retailers and governments, to increase demand for healthier food (and decrease demand for unhealthy food) were also identified, particularly through strategies that supported an even playing field across the food industry.

Participants spoke about the variety of ways the food industry increased consumer demand for less healthy food through the products they make, the trends they create or respond to and their marketing practices, including pricing, package design, nutrition labelling and promotions. These reflections aligned with the Consumer Demand Cycle presented to them (Figure 1). Participants’ acknowledgement of food companies’ responsibility for shaping consumer demand contrasted with the public narrative often presented by the food industry which deflects industry responsibility for unhealthy population diets and emphasises individual responsibility for diet choices and physical activity^(17–19,29,37)^. Nonetheless, a focus on individual responsibility was also evident in this study, where food companies framed their actions as simply responding to consumer demand for affordable foods that children are willing to eat, and for premium or indulgent foods that offer joy and comfort.

Our study found that perceived consumer demand for indulgent foods led companies, in many instances, to prioritise taste over health, which meant certain products were often exempt from reformulation efforts. This is consistent with research that found soft drink marketing and reformulation strategies were substantially influenced by the perceived brand strength and identity of the products and consumer trends^(38)^. This selective approach to reformulation may undermine efforts to improve population diets if many products that contribute significantly to excess sugar, salt and saturated fat intake remain unaddressed. Nevertheless, several companies in this study indicated that their company had adopted a stepwise approach to reformulating less healthy products to minimise potential consumer backlash. Developments in food technology, such as more widespread use of salt substitutes, may provide further opportunities for reformulation for health benefit without compromising taste or risking consumer backlash^(39)^.

Participants also recognised broader elements and drivers that influenced consumer demand, such as the promotion, placement and distribution tactics of retailers. These findings align with research showing that retailer innovations and marketing strategies, such as merchandising, pricing and promotions, play a key role in shaping consumer preferences and demand^(40)^. Importantly, research indicates that large food manufacturers and major retailers (e.g. large supermarket chains) typically work together to shape the selection, placement, marketing and pricing of products within retail environments^(41,42)^.

This study found that the competitive landscape, that is, food companies’ drive to gain or maintain market advantage, was cited as a barrier to increasing demand for healthy food and reducing demand for unhealthy products. Similar concerns were expressed in another study, where food industry representatives discussed reluctance to improving the healthiness of supermarket price promotions due to a fear of losing competitive advantage^(42)^. It was clear that companies were unlikely to voluntarily implement healthier changes that risk a reduction to their profits, particularly if it was thought that consumers might switch to a competitor’s product instead. While other work has highlighted the potential for the food industry, and their investors, to profit from shifts to healthier products, perceived risks related to potential loss of profits from doing so were predominant in our study^(21,43)^. These findings align with research that has found industry self-regulation and voluntary nutrition initiatives have poor uptake and are largely ineffective^(44–47)^. Similarly, a German study found limitations related to the competitive landscape in relation to supermarkets’ ability to drive transformative change towards sustainability in the food system, with stakeholders citing ‘dysfunctional markets’ that prioritised profits as a major obstacle^(48)^.

Opportunities to influence consumer demand in ways that support healthier population diets identified by participants included food companies engaging their marketing teams in nutrition goals to encourage strategies that promote public health and calling on retailers to prioritise the availability, affordability and promotion of healthy over unhealthy foods. Our study also found that participant perspectives about what is needed to shift consumer demand differed in some instances from those often discussed publicly. For example, many company representatives expressed support for mandatory implementation of the HSR system, viewing it as a way to create a level playing field for food companies and enable consumers to make more informed choices about food healthiness. This position contrasted with the industry’s documented preference for self-regulation and that voluntary industry actions are sufficient to address the healthiness of the food supply^(16–18)^. This shift may reflect growing awareness among food company representatives of international trends towards regulatory measures in high-income countries and aligns with evidence that industry actors increasingly view such regulations as opportunities to create a level playing field^(49)^. Studies that have benchmarked the nutrition-related policies and actions of Australian food companies have found considerable opportunity for improvement, with recommendations that include setting and reporting on healthy and unhealthy food sales targets; reducing children’s exposure to unhealthy food marketing; fully implementing and reporting progress on the HSR system; and working with retailers to ensure healthy products are affordable, accessible and prioritised in promotions^(13,14)^. However, in the absence of stronger food industry action to drive consumer demand for healthy products, government regulations, such as mandatory front-of-pack nutrition labelling and restrictions on unhealthy food marketing, with ongoing independent monitoring of food company policies and practices, are crucial.

### Strengths and limitations

This is the first study to explore perspectives from food industry representatives on how industry actions have driven consumer demand for unhealthy diets. A key strength of the study was that it included company representatives with a wide variety of roles in different food companies, including varying levels of expertise and experience in relation to nutrition. Representatives involved were from companies who voluntarily agreed to participate in the REFORM study, which, because of the nature of the intervention programme, may have contributed to selection bias towards companies with greater interest in nutrition and health. These companies may be more receptive to industry changes that support healthier products and food choices. Perspectives of company representatives responding to the research team questions may have been influenced by social acceptability bias and skewed responses to be more supportive of greater action on nutrition from the food industry. While the research team made deliberate efforts to manage group dynamics and encourage engagement from all company representatives, the group setting may have led to the disproportionate influence of more senior or dominant voices, and it is unclear whether the views of the people we spoke to represent the views of others within the company. Future research should engage a broader spectrum of food companies, particularly small businesses who may have different perspectives, and extend to other industry sectors like retailers and fast-food chains. Finally, relying on written notes rather than audio recordings may have limited the capture of participants’ tone, language and nuanced framing.

## Conclusions

This study found that food companies acknowledge that they play an active role in driving demand for both healthy and unhealthy food and identified several ways in which they seek to influence consumer choice. These findings contradict common company arguments that they merely respond to consumer demand, and that dietary choices are an individual responsibility. Some food companies in this study perceived profit-related risks in focusing on health and nutrition in particular food categories served as a barrier to more nutrition-related action. In this way, the findings strengthen public health-related arguments for structural changes to the food supply and the characteristics of food environments as part of efforts to improve population diets. In particular, regulatory changes are likely to be needed to create an even playing field that can support food companies to increase demand for healthier diets. The study indicated that such regulatory changes are likely to be welcomed by at least some major food companies. At the same time, governments and the public health community can work together to provide clear and consistent nutrition policy and practice recommendations for food companies to implement, whilst holding food companies accountable for implementing these recommendations.

## Supporting information

Marshall et al. supplementary material 1 Marshall et al. supplementary material

Marshall et al. supplementary material 2 Marshall et al. supplementary material

Marshall et al. supplementary material 3 Marshall et al. supplementary material

## Supplementary material

For supplementary material accompanying this paper visit https://doi.org/10.1017/S1368980026101943
