# Supplementary material for: The food industry’s role in influencing consumer demand for healthy and unhealthy food: perspectives from Australian food companies
Source: Public Health Nutr. 2026 Jan 30;29(1):e33. doi: 10.1017/S1368980026101943 (PMC12917402; doi:10.1017/S1368980026101943)
Supplement: Marshall et al. supplementary material 1 — Marshall et al. supplementary material [file S1368980026101943sup001.pdf]

reform
Supporting Companies to Reform Nutrition Policies and Practices

GLOBE GLOBAL OBESITY CENTRE · INSTITUTE FOR HEALTH TRANSFORMATION · DEAKIN UNIVERSITY · NIHI The National Institute for Health Innovation · THE UNIVERSITY OF AUCKLAND NEW ZEALAND

Touchpoint 3: Consumer Insights

Company name

Date

1

The REFORM program

Our aim is to provide **tailored support** to the largest food companies in Australia to improve **nutrition-related policies and practices**

Touchpoint	Focus
1	**Food Composition**
2	**Nutrition Labelling**
3	**Consumer Insights** ←
4	**Nutrition Policy Assessment and Sustainability Reporting**
5	**Industry Recognition Scheme**
	**Program Wrap Up**<br>**Post-Program Follow Up**

2

Agenda

1. Updates since last meeting
2. Influences on consumer demand for food
3. Emerging nutrition trends
    - Sustainability and concerns for planetary health
    - Clean label and whole foods
    - Reducing sugar and sweeteners
    - Maximising gut health and fibre intake
    - Permissible indulgence
4. Next steps

Follow up from last meeting

- Feedback from previous meeting on Nutrition Labelling
    - Meeting slides, PDF resource
    - Have they been useful?
    - Any further questions?
- Any other nutrition-related updates?
- CSIRO support

Nutrition more than a ‘trend’ – moving towards being a core focus for the food industry

The FAO urges food industry to do more to support healthy foods

The FAO’s Director-General calls on the food industry to transform production and supply and value chains.

Megatrends: Quantifying Wellness

10/7/2021

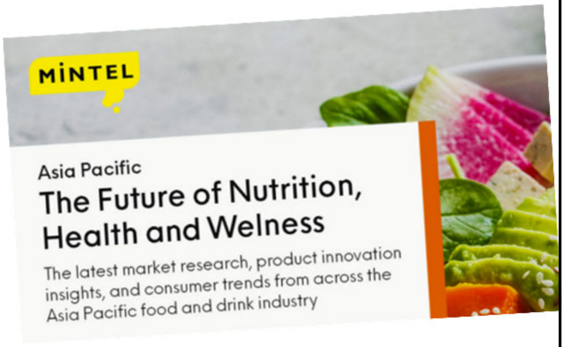

DIVE BRIEF

Consumers are more confident about healthy eating as pandemic impacts wane, study says

Published May 19, 2021

Consumer trends shifting toward health and wellness, ADM finds

The pandemic has made more people interested in foods that benefit their immunity, metabolism and mental state, as well as solidified the plant-based sector, according to research.

Published Sept. 3, 2020

What role should the commercial food system play in promoting health through better diet?

OPEN ACCESS

Martin White and coauthors consider that the commercial food system has the potential to show leadership and support for dietary public health, but systemic change is needed first and this is likely to require governmental action

5

Many major companies are embracing demand for healthy foods

Birds Eye exec flags significant shift in consumer desire for healthy food over last decade

– A +

19 Jan 2022 --- International frozen foods giant Birds Eye is stressing that a ramped-up focus on nutrition is a “golden opportunity” for food brands, following a report that reveals a strong consumer desire to eat better.

Cutting down on salt, sugar and fat is a crucial part of the company’s analysis, which zeroes in on how government-led strategies are targeting the obesity crisis. The report also underscores a lack of consumer knowledge about saturated and unsaturated fat.

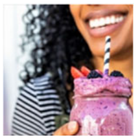

Unilever sets healthy food course in wake of criticism for lagging peers

Unilever will measure its performance against six nutrient profile models, including NutriScore.

healthy life food tracker

POWERED BY

Woolworths | everyday rewards

Woolworths are offering customers a breakdown of their food purchases according to the Australian Dietary Guidelines.

The balance of your shop:

Your plate shows a % balance of your shopping broken down by food group and compares that to Australian Dietary Guidelines.

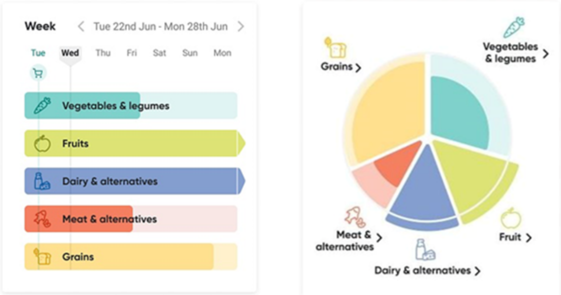

6

Consumers want to eat healthier diets

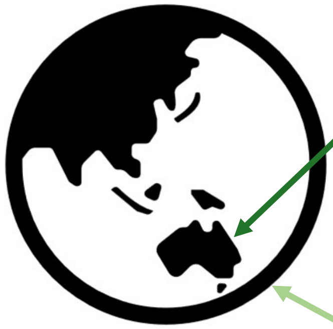

89% of Australians want a healthier diet. (Davidson Branding 2021 - The Future of Healthy Food)

61% of Australians are actively trying to eat healthy. (Statista 2021 update 2), Global Consumer Survey, Australia)

70% of Australians think food companies have a responsibility to make foods healthier. (International Food Policy Study 2021, preliminary results)

- **Globally,** half of consumers seek health & nutritional properties. (Euromonitor 2021 - The Impact of Third-party Nutrition Labelling)
- **In the UK**, 66% of people are thinking about or already changing their diets, and they want to be supported. (IGD 2021 – Appetite for Change)

Beyond consumer interest in healthy eating, **everyone has the right to healthy, safe food.**
(FAO 2022)

However, Australians diets are not in line with the Australian Dietary Guidelines

Sales & consumption data show Australian diets **are below** the recommendations across all the core five food groups.

38% of total energy intake from food and non-alcoholic beverage sales are from discretionary products

ABS Australian Health Survey 2011-12

Daily per capita serves of ADG five food groups(a)

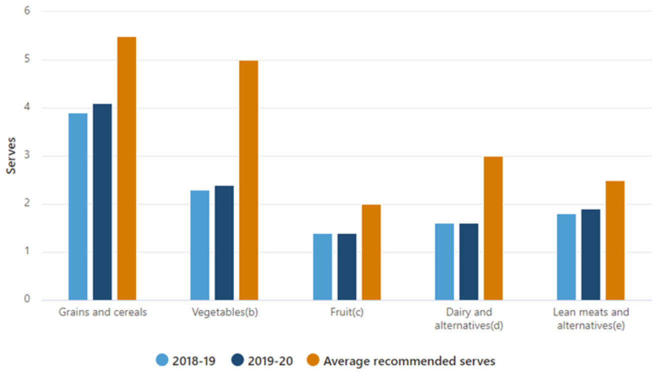

a. Includes non-discretionary foods only.
b. Vegetables and legumes/beans.
c. Includes fruit juice.
d. Milk, yoghurt, cheese and/or alternatives.
e. Lean meats, poultry, fish, eggs, tofu, nut and seeds and legumes/beans.

ABS Apparent Consumption of Selected Foodstuffs 2019-20

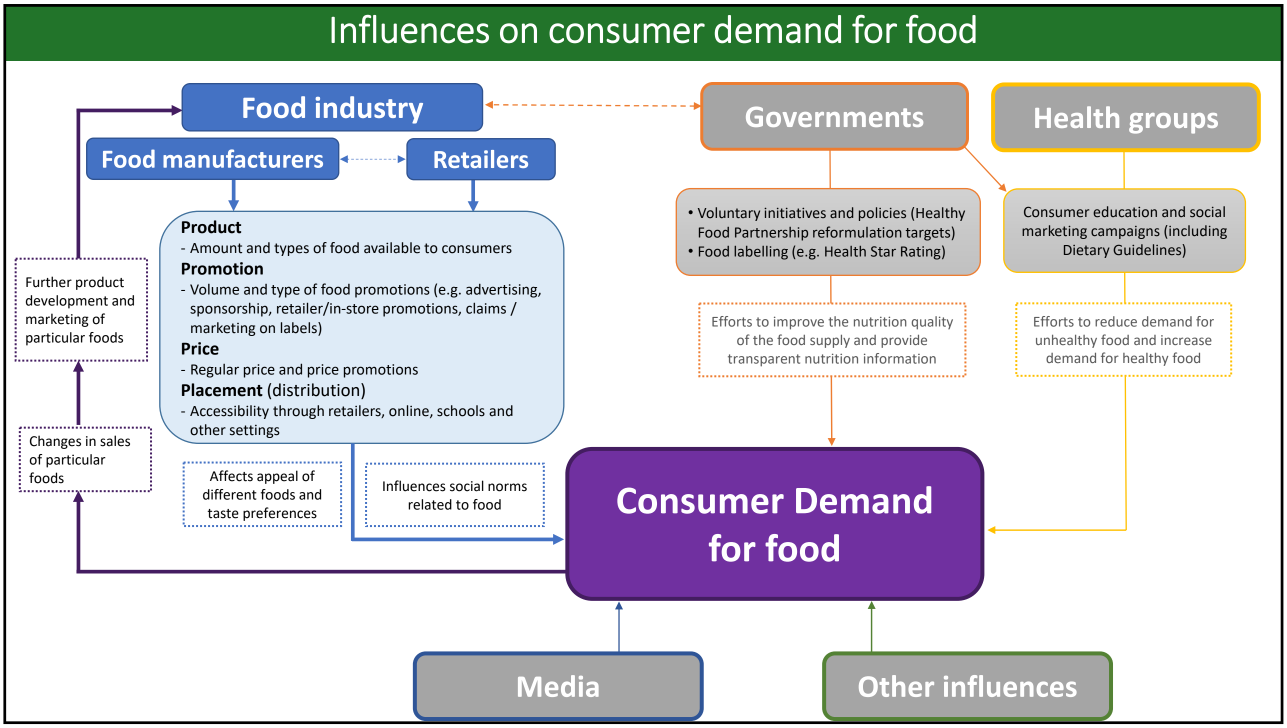
Influences on consumer demand for food
Food industry
Food manufacturers
Retailers
Governments
Health groups
Product
- Amount and types of food available to consumers
Promotion
- Volume and type of food promotions (e.g. advertising, sponsorship, retailer/in-store promotions, claims / marketing on labels)
Price
- Regular price and price promotions
Placement (distribution)
- Accessibility through retailers, online, schools and other settings
• Voluntary initiatives and policies (Healthy Food Partnership reformulation targets)
• Food labelling (e.g. Health Star Rating)
Consumer education and social marketing campaigns (including Dietary Guidelines)
Efforts to improve the nutrition quality of the food supply and provide transparent nutrition information
Efforts to reduce demand for unhealthy food and increase demand for healthy food
Further product development and marketing of particular foods
Changes in sales of particular foods
Affects appeal of different foods and taste preferences
Influences social norms related to food
Consumer Demand for food
Media
Other influences

9

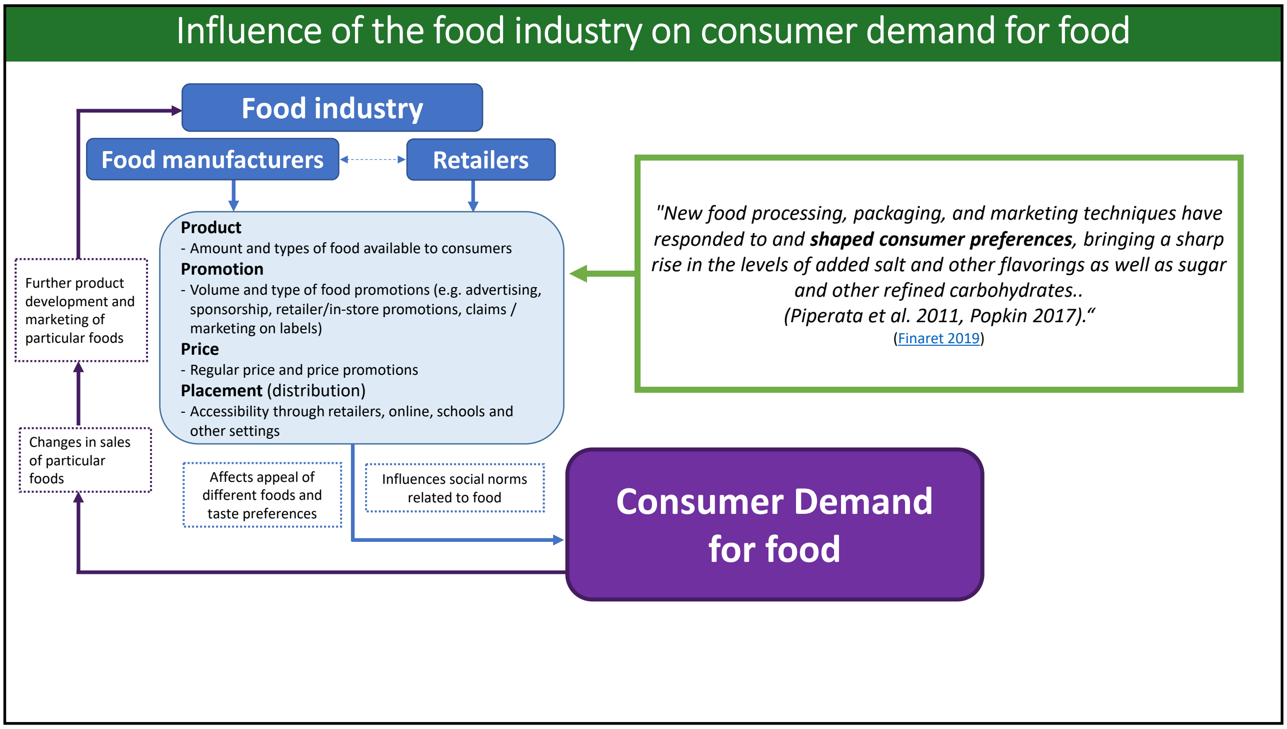
Influence of the food industry on consumer demand for food
Food industry
Food manufacturers
Retailers
Product
- Amount and types of food available to consumers
Promotion
- Volume and type of food promotions (e.g. advertising, sponsorship, retailer/in-store promotions, claims / marketing on labels)
Price
- Regular price and price promotions
Placement (distribution)
- Accessibility through retailers, online, schools and other settings
"New food processing, packaging, and marketing techniques have responded to and shaped consumer preferences, bringing a sharp rise in the levels of added salt and other flavorings as well as sugar and other refined carbohydrates.. (Piperata et al. 2011, Popkin 2017)."
(Finaret 2019)
Further product development and marketing of particular foods
Changes in sales of particular foods
Affects appeal of different foods and taste preferences
Influences social norms related to food
Consumer Demand for food

10

Once ‘healthy’ product categories have evolved to become more processed and less healthy

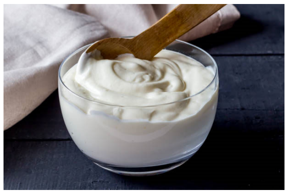

Plain unsweetened yoghurt

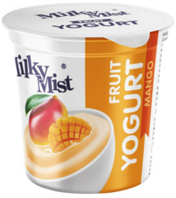

Sweetened yoghurt

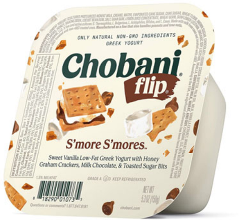

Marshmallow flavoured yoghurt, cookie crumble, milk chocolate, coated rice crisps

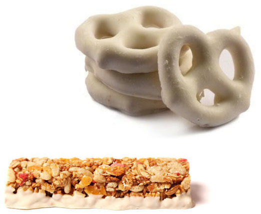

[illegible]

Core, whole food → ultra-processed, discretionary products

One in five consumers are not sure what foods are considered healthy

Euromonitor 2021 - The Impact of Third-party Nutrition Labelling

Consumers want transparent nutrition labelling

*“**Clear and accurate nutrition labelling is now essential** as the role of healthy diets in preventing certain health conditions becomes top of mind for consumers.”*

(*Euromonitor 2020 - Wellness Redefined: Healthy Eating in a Post-Coronavirus World*)

80% of Australian consumers think food companies should clearly display the HSR on all packaged food and drinks.
(International Food Policy Study 2021, preliminary results)

62% of Australian consumers think food companies should only make nutrition claims on products that are healthy overall (International Food Policy Study 2021, preliminary results)

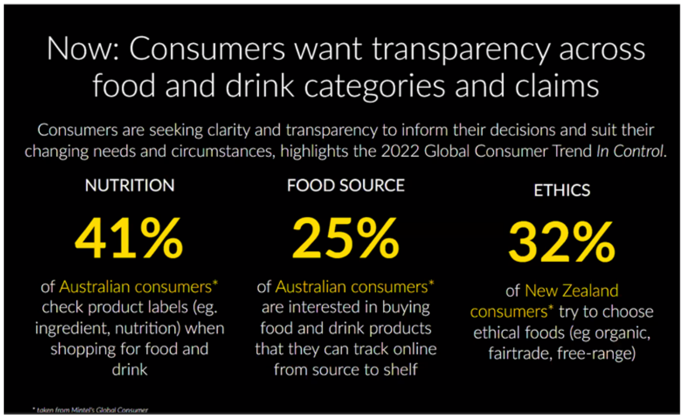

Mintel webinar 2022: Food & drink applications of the 2022 consumer trends: Australia and New Zealand

Reducing risk nutrients & increasing whole foods are key concerns for consumers

Lowering risk nutrients top of mind for Australian parents when shopping for children's packaged food

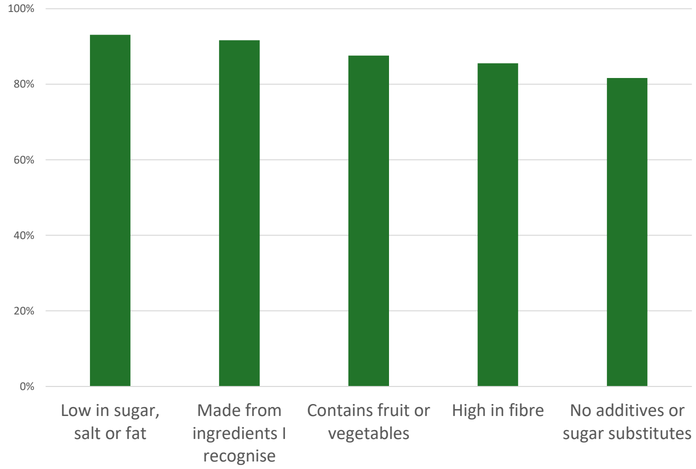

Parents Voice 2021 survey of 324 Australian parents (Preliminary results)

Also reflected globally

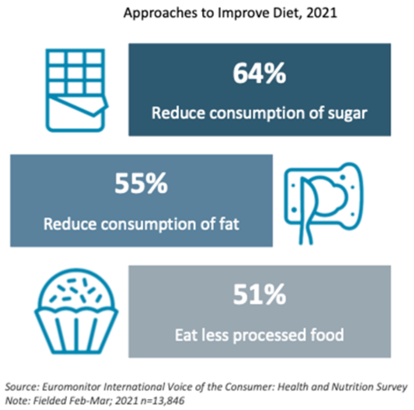

Euromonitor 2021, The Evolution of the Eating Occasion

Parents want existing products to be healthier

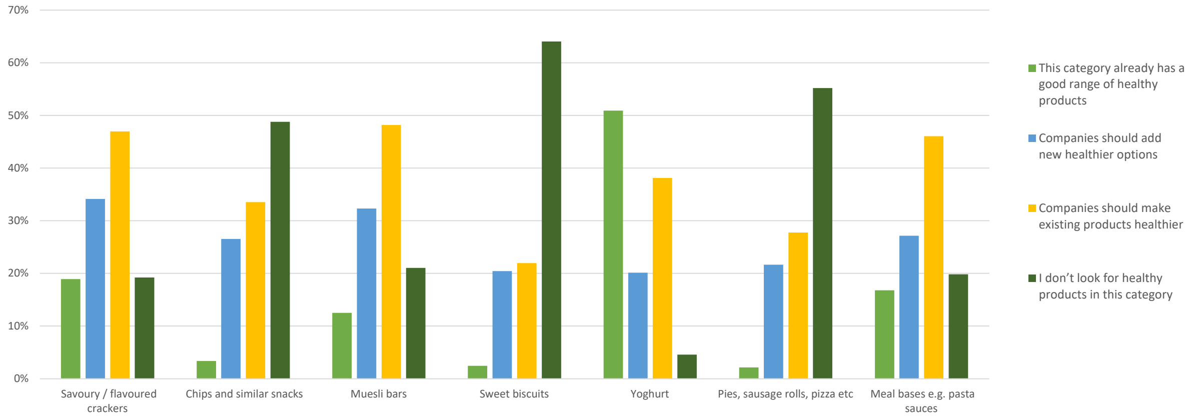

Participants were asked: Which statement best describes what you think about the products available in each of these food categories? (choose all that apply)

Parents Voice 2021 survey of 324 Australian parents (Preliminary results)

Emerging trends provide opportunities for health

1. Sustainability & concerns for planetary health

2. Push for clean label & whole foods

3. Reducing sugar & sweeteners

4. Maximising gut health & fibre intake

5. 'Permissible indulgence'

1. Sustainability needs to underpin our food system

The plant-based trend appeals to consumers concerned about planetary health, health and nutrition, and animal welfare.

23% consumers globally are trying to limit their meat intake

16% are following a plant-based diet

(Euromonitor 2021 - Plant-based Eating and Alternative Proteins)

19%

of Australians are reducing their meat consumption

Malek 2021

Diets in Australia:

- 9% flexitarian
- 5% vegetarian
- 3% pescatarian
- 2% vegan
- 11% would consider adopting a fully vegan diet in the next 5 years

Choice 2021

Plant-based diet: benefits health and sustainability

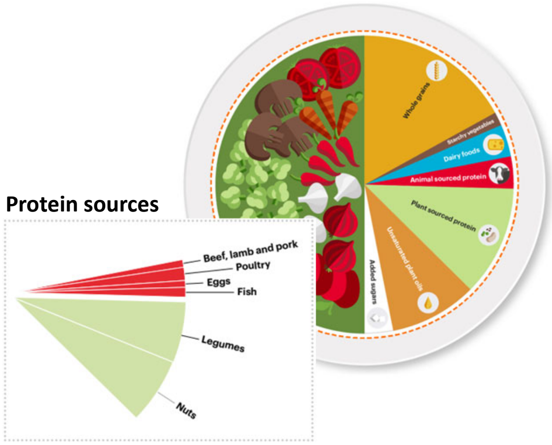

EAT-Lancet Commission on Food, Planet, Health

Industry response to plant-based trend has resulted in more ultra-processed, unhealthy food

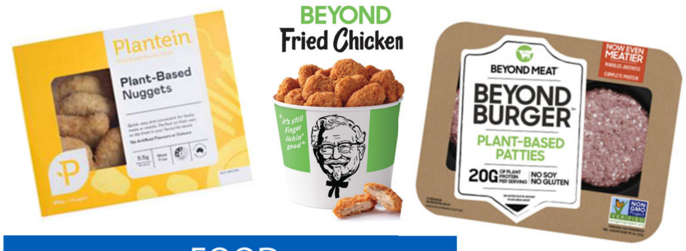

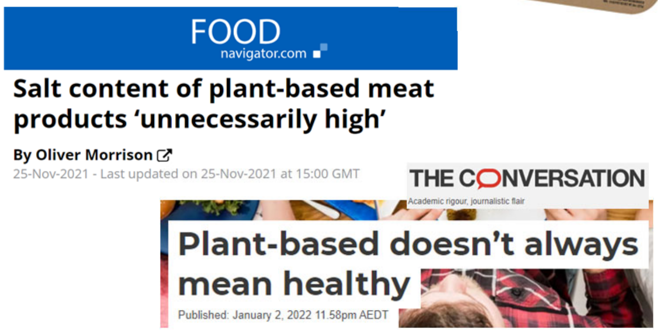
FOOD navigator.com

Salt content of plant-based meat products 'unnecessarily high'

By Oliver Morrison

25-Nov-2021 - Last updated on 25-Nov-2021 at 15:00 GMT

THE CONVERSATION

Academic rigour, journalistic flair

Plant-based doesn't always mean healthy

Published: January 2, 2022 11.58pm AEDT

Recommendations:

- Focus efforts to incorporate plant-based foods on including **unprocessed, whole food ingredients** such as fruits, vegetables, nuts and legumes.
- Ensure **risk nutrients** (sugar, saturated fat, sodium) **are limited** in plant-based products.

2. Consumers want to eat more whole foods and recognise the ingredients they're eating

'Clean label' food refers to food or drink products made from a small number of natural, wholesome, easily recognisable ingredients.
Choice 2021

81% of global consumers believe short and simple ingredient lists are important (Davidson Branding 2021 - The Future of FMCG)

82% of global consumers want to recognise the ingredients in packaged foods, rather than those that sound like chemicals (Davidson Branding 2021 - The Future of FMCG)

64% of Australians want to purchase products with natural ingredients (IRI 2021 – State of the Industry)

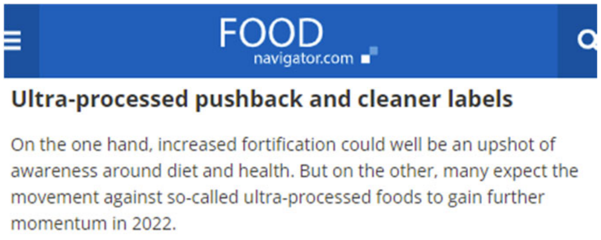
FOOD navigator.com

Ultra-processed pushback and cleaner labels

On the one hand, increased fortification could well be an upshot of awareness around diet and health. But on the other, many expect the movement against so-called ultra-processed foods to gain further momentum in 2022.

Recommendations:

- Focus reformulation efforts and new product development on using **whole food ingredients and reducing ultra-processed foods**.
- **Prioritise 'clean' products over clean labels**, i.e. rather than renaming/deleting an ingredient in the ingredients list, consider removing it entirely from the product formulation.

3. Consumers want foods that support a healthy gut

- A healthy diet is associated with benefits beyond digestive health, such as general health, chronic disease prevention and mental health. (Deakin University Food and Mood Centre)
- Gut health is a priority for **55%** of Australians. (Food & Beverage Industry News 2021, research commissioned by Kelloggs)
- Almost **three in five** US adults are trying to eat foods that support a healthy gut. (Mintel 2020 - The Future Foundation of a Healthy Gut)

Recommendations:

- ❑ **Utilise whole foods** such as wholegrains, legumes, nuts, fruits, vegetables to increase fibre content in products, rather than using isolated, highly processed fibres.
- ❑ **Avoid 'health-washing'**: e.g. ensure probiotics are not added to otherwise unhealthy products, ensure the overall healthiness of products with added probiotics or gut health claims.

"All disease begins in the gut"

Greek physician Hippocrates, nearly 2500 years ago

FOOD navigator.com

Consumers increasingly 'making the link between gut health and overall health'

By Oliver Morrison

18-Aug-2021 - Last updated on 18-Aug-2021 at 15:20 GMT

Immune and gut health: why 1 in 4 global consumers are making it a priority

4. Australians want to reduce sugar intake

- **82%** Australian consumers want to decrease their sugar intake (Davidson Branding 2021 - The Future of Healthy Food)
- **52%** of global consumers believe reducing sugar intake would help them feel healthier (Euromonitor 2021 - Beverage Manufacturers Drive Reformulation Efforts Through Sugar Reduction)
- Consumers report avoiding 'artificial' sweeteners, preferring products that are 'natural' (Nunn 2021)

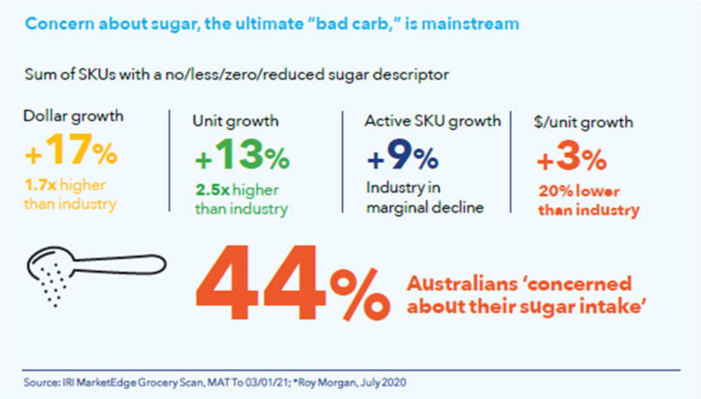

IRI 2021 A Healthier New Normal

Recommendations:

- ❑ **Reduce added sugar** in existing products and limit in new product development
- ❑ **Reduce the overall sweetness** of products
- ❑ **Limit the use of sweeteners** as a replacement for sugar

5. “Permissible indulgence” trend - these products are still discretionary foods

- The “permissible indulgence” trend attempts to merge consumer concerns about health while delivering on taste and indulgence.
- Despite the different terminology, these are often unhealthy, discretionary foods.

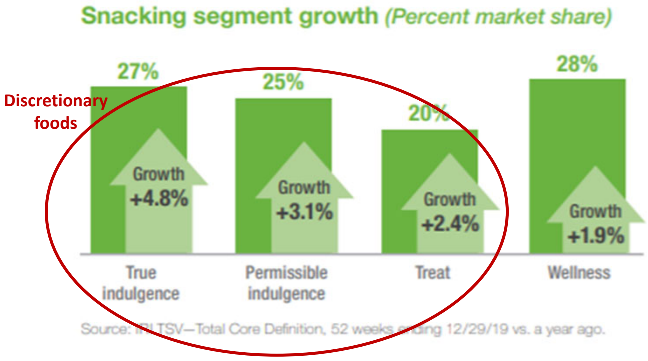

Cargill 2021 - Snacking in 2020: A Year for the Record Books

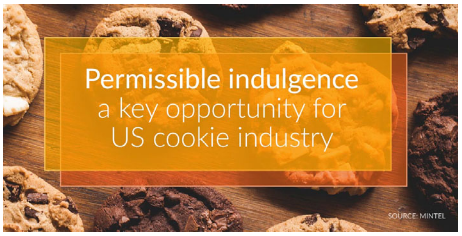

Mintel 2020 - Permissible indulgence a key opportunity for US cookie industry

Some products in discretionary categories are now marketed as healthy

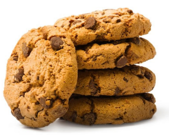

Sweet biscuits

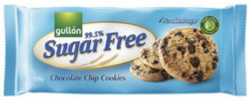

Reduced sugar biscuits, with sweeteners

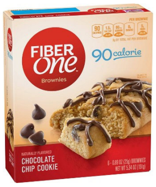

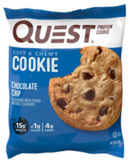

Biscuits with added fibre/protein

Discretionary products →
slightly healthier, but still discretionary products

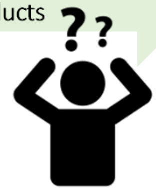

Contributes to consumer confusion of what is considered healthy and discretionary

Recommendations:

- ❑ Despite improvements from an original “indulgent” food, these products **should not be marketed as healthy** if they are discretionary foods.
- ❑ Ensure unhealthy/“indulgent” foods are **a smaller part of your overall portfolio mix.**
- ❑ Include “indulgent” / premium products in **reformulation targets and nutrition reporting.**

Influence of the food industry on consumer demand for food

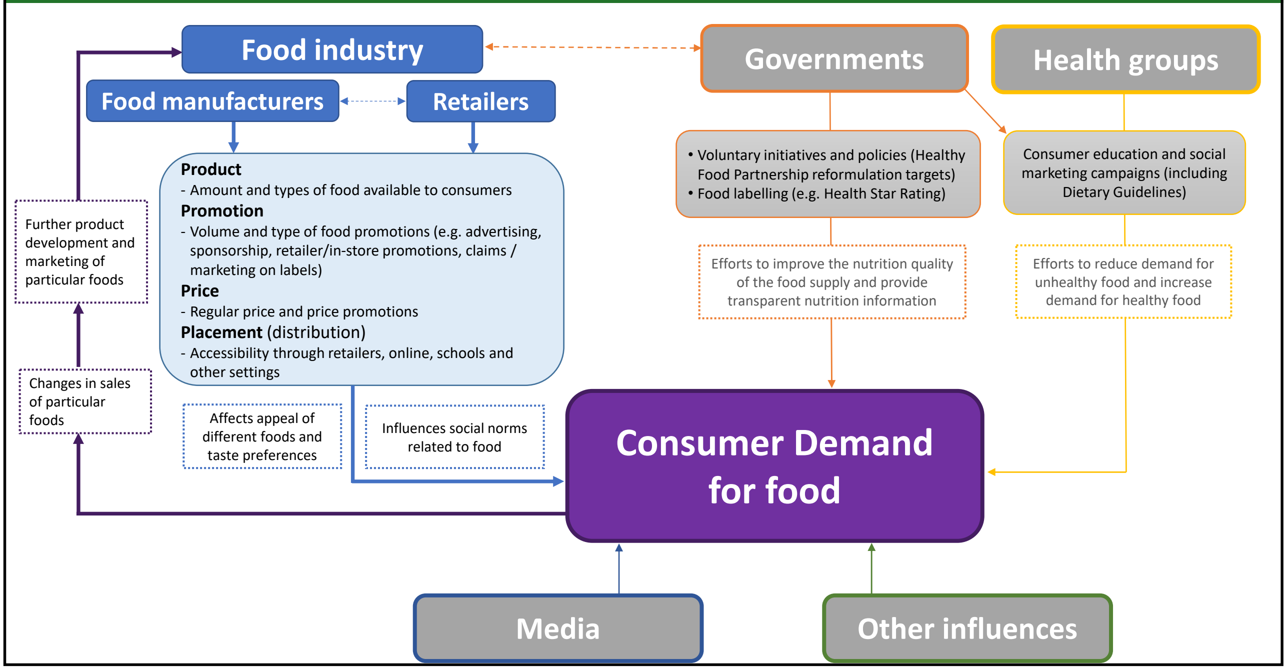

23

Strong role for industry to authentically meet consumer demand for healthier diets

Summary recommendations:

- ❑ Integrate nutrition as a core part of your business.
- ❑ Future proof your portfolio to reflect healthier foods that benefit people and the planet.
- ❑ Take a holistic approach when incorporating consumer trends:
  - ❑ Focus reformulation efforts and new product development on reducing risk nutrients.
  - ❑ Using whole food ingredients such as fruit, vegetables, nuts, seeds, legumes, and wholegrains, and look for opportunities to limit processing.
- ❑ Authentically communicate product healthiness:
  - ❑ Provide transparent nutrition information.
  - ❑ Ensure marketing/branding supports consumers to meet their healthy eating goals.
- ❑ Reduce risk to the business by considering short term changes as part of a long term strategy.

> *"Successful companies will be those that improve the health of the planet and population."*

[Mintel 2020 Global Food and Drink Trends 2030]

24

Next steps

- REFORM team to send resources
- Schedule next meeting: *Nutrition Policy and Corporate Social Responsibility*

Any questions?

Research team contact details

The content of this resource is general in nature and, although based on the referenced information, it is recommended you refer to the source documents or websites for the most up to date and accurate information. Companies should make their own assessment of the information presented and seek further guidance, as needed, prior to making any business decisions.
