# Supplementary material for: The food industry’s role in influencing consumer demand for healthy and unhealthy food: perspectives from Australian food companies
Source: Public Health Nutr. 2026 Jan 30;29(1):e33. doi: 10.1017/S1368980026101943 (PMC12917402; doi:10.1017/S1368980026101943)
Supplement: Marshall et al. supplementary material 2 — Marshall et al. supplementary material [file S1368980026101943sup002.docx]

**Appendix B: Interview Guide**

Interview question prompts:

- What are your initial thoughts on this [flowchart, see Figure 1]?
  - Is this relevant to your company/do you see this in your company or have something similar?
- Are you aware of your role in driving consumer demand?
  - What's your role in shaping consumer demand?
- What are you hearing from consumers?
  - Where do you get this info from?
  - Do you have a global or national consumer insights team or do you look to market research companies?
  - What role does marketing play in consumer insights?
- How can you support consumer demand for healthy food and shift people to healthier eating patterns?
  - 4 P’s (product, promotion, price, placement)
  - How do you see the pulling of these levers?
  - What helps support these changes?
  - What are some of the barriers to progress?
  - Thinking about short term, as well as long term actions?
- What is the role of the retailer and the dynamic with manufacturers in shaping/responding to demand?
- What support do you want from other influences (government, public health groups, etc.)?
- What broader changes to the system would support change?
  - For example, we’ve heard companies speak about issues with other companies using healthy food marketing on unhealthy food that changes their marketing approach, or having to respond to pressure from the retailer.
- Is there anything else you’d add to this flowchart?
