# Supplementary material for: The food industry’s role in influencing consumer demand for healthy and unhealthy food: perspectives from Australian food companies
Source: Public Health Nutr. 2026 Jan 30;29(1):e33. doi: 10.1017/S1368980026101943 (PMC12917402; doi:10.1017/S1368980026101943)
Supplement: Marshall et al. supplementary material 3 — Marshall et al. supplementary material [file S1368980026101943sup003.docx]

Appendix C: COREQ (COnsolidated criteria for REporting Qualitative research) Checklist

Developed from: Tong A, Sainsbury P, Craig J. Consolidated criteria for reporting qualitative research (COREQ): a 32-item checklist for interviews and focus groups. *International Journal for Quality in Health Care*. 2007. Volume 19, Number 6: pp. 349 – 357

| **Topic** | **Guide Questions/Description** | **Response/Details from the manuscript** | **Location in manuscript** |  |
| --- | --- | --- | --- | --- |
| **Domain 1: Research team and reﬂexivity** | | | | |
| Personal characteristics | | | | |
| 1. Interviewer/facilitator | Which author/s conducted the interview or focus group? | “2-3 research team members (JM, JC, SD)” | Methods, line 132 |  |
| 1. Credentials | What were the researcher’s credentials? E.g. PhD, MD | “two professors with PhDs, three associate research fellows (BNutrDiet(Hons), BNut(Hons), BHealthSci) and one PhD candidate (BHealthSci(Hons))” | Methods, line 191-192 |  |
| 1. Occupation | What was their occupation at the time of the study? | “all full-time public health researchers at universities” | Methods, line 190-191 |  |
| 1. Gender | Was the researcher male or female? | “The research team included five women and one man” | Methods, line 190 |  |
| 1. Experience and training | What experience or training did the researcher have? | “all full-time public health researchers at universities, two professors with PhDs, three associate research fellows (BNutrDiet(Hons), BNut(Hons), BHealthSci) and one PhD candidate (BHealthSci(Hons)).”  “The research team has extensive expertise in policy research to improve population diets and public health and shares the view that a whole of system approach is required to improve the healthiness of the food environment, involving both private sector and government action” | Methods, line 190-192 and 178-181 |  |
| Relationship with participants | | | | |
| 1. Relationship established | Was a relationship established prior to study commencement? | “The research team had limited (GS) or no (JM, JC, SS, SD, CNM) prior relationship with the company representatives before the series of meetings.” | Methods, line 129-131 |  |
| 1. Participant knowledge of   the interviewer | What did the participants know about the researcher? e.g. personal goals, reasons for doing the research | “research team members introduced themselves during meetings, including their occupation and role in the study” | Methods, line 135-136 |  |
| 1. Interviewer characteristics | What characteristics were reported about the interviewer/facilitator? e.g. Bias, assumptions, reasons and interests in the research topic | “Participants were informed of the purpose of the REFORM study and research team members introduced themselves during meetings, including their occupation and role in the study.” | Methods, line 134-136 |  |
| **Domain 2: Study design** | | | | |
| Theoretical framework | | | | |
| 1. Methodological orientation and Theory | What methodological orientation was stated to underpin the study? e.g. grounded theory, discourse analysis, ethnography, phenomenology,  content analysis | “Data were analysed thematically using Braun and Clarke’s 6-step approach” | Methods, line 165-166 |  |
| Participant selection | | | | |
| 1. Sampling | How were participants selected? e.g. purposive, convenience, consecutive, snowball | Purposive sampling was used:  “REFORM focused on the largest packaged food and beverage manufacturers in Australia and New Zealand (>$10 million in annual retail sales revenue), identified using Euromonitor 2019 data and randomised to intervention and control groups” | Methods, line 84-86 |  |
| 1. Method of approach | How were participants approached? e.g. face-to-face, telephone, mail, email | “invited to be part of the REFORM program via email and/or phone calls,” | Methods, line 97-98 |  |
| 1. Sample size | How many participants were in the study? | “13 companies with head offices in Australia accepting and participating in the program.” | Methods, line 98 |  |
| 1. Non-participation | How many people refused to participate or dropped out? Reasons? | 34 companies declined to participate or did not respond. No companies dropped out.  “Forty-seven companies were invited to be part of the REFORM program…with 13 companies…accepting and participating in the program” | Methods, line 97-98 |  |
| Setting | | | | |
| 1. Setting of data collection | Where was the data collected? e.g. home, clinic, workplace | Meetings were held online using Zoom or Microsoft Teams” | Methods, line 133 |  |
| 1. Presence of non-participants | Was anyone else present besides the participants and researchers? | No - “Each of the 13 separate meetings on this topic involved 2-3 research team members (JM, JC, SD) and representatives from within the food company.” | Methods, line 131-133 |  |
| 1. Description of sample | What are the important characteristics of the sample? e.g. demographic data, date | Refer to methods – “Characteristics of participating companies and representatives” and Table 1 | Methods, line 96-125 and Table 1 |  |
| Data collection | | | | |
| 1. Interview guide | Were questions, prompts, guides provided by the authors? Was it pilot tested? | “company representatives were asked for their views on the framework and how they perceived their company influenced consumer demand for healthy and unhealthy food (approximately 40 minutes). See **Appendix B** for the semi-structured interview guide.” | Methods, line 143-146 and Appendix B |  |
| 1. Repeat interviews | Were repeat interviews carried out? If yes, how many? | n/a | n/a |  |
| 1. Audio/visual recording | Did the research use audio or visual recording to collect the data? | “Detailed written field notes including verbatim responses were taken by a dedicated member of the research team (SD), with permission from the company representatives. Meetings were not audio recorded,” | Methods, line 148-150 |  |
| 1. Field notes | Were ﬁeld notes made during and/or after the interview or focus group? | “Detailed written field notes including verbatim responses were taken by a dedicated member of the research team”  “Field notes were reviewed immediately after each meeting by the other 1-2 members of the research team” | Methods, line 148-153 |  |
| 1. Duration | What was the duration of the interviews or focus group? | “average duration of 60 minutes.” | Methods, line 134 |  |
| 1. Data saturation | Was data saturation discussed? | No | n/a |  |
| 1. Transcripts returned | Were transcripts returned to participants for comment and/or correction? | “To maintain analytic independence and avoid potential industry influence on how findings were interpreted, company representatives were not given field notes or draft themes to review.” | Methods, line 154-156 |  |
| **Domain 3: analysis and ﬁndings** | | | | |
| Data analysis | | | |  |
| 1. Number of data coders | How many data coders coded the data? | “field notes for two companies being coded independently by three researchers (JM, JC, SS)”  “Two researchers (JM, JC) then used this coding framework to deductively code the remaining data, independently” | Analysis, line 167-172 |  |
| 1. Description of the coding tree | Did authors provide a description of the coding tree? | No | n/a |  |
| 1. Derivation of themes | Were themes identiﬁed in advance or derived from the data? | “draft themes were constructed from the codes by the first author (JM), then presented to members of the research team (JC, SS, SD, GS) where the themes were reviewed and workshopped” | Analysis, line 173-175 |  |
| 1. Software | What software, if applicable, was used to manage the data? | “Data (field notes) were managed using QSR N-Vivo (Release 1.7).” | Analysis, line 165 |  |
| 1. Participant checking | Did participants provide feedback on the ﬁndings? | “To maintain analytic independence and avoid potential industry influence on how findings were interpreted, company representatives were not given field notes or draft themes to review.” | Methods, line 154-156 |  |
| Reporting | | | |  |
| 1. Quotations presented | Were participant quotations presented to illustrate the themes/ﬁndings?  Was each quotation identiﬁed? e.g. participant number | Yes, quotations used and identified e.g. “(Manager (Marketing), Company 10-043)” | Results, from line 211 |  |
| 1. Data and ﬁndings consistent | Was there consistency between the data presented and the ﬁndings? | Yes, as supported by the quotes within each theme. | Results, from line 211 |  |
| 1. Clarity of major themes | Were major themes clearly presented in the ﬁndings? | Yes, six themes were presented in the results, e.g. “Theme 1: Food manufacturers acknowledge their role in creating demand for healthy and unhealthy food” | Results, from line 211 |  |
| 1. Clarity of minor themes | Is there a description of diverse cases or discussion of minor themes? | Yes, within each theme, any contrasting views were presented. | Results, from line 211 |  |
